# Protein kinase R regulates pancreatic ductal adenocarcinoma progression by modulating the cell cycle via *GADD45A*

**DOI:** 10.1038/s41598-025-06213-4

**Published:** 2025-07-10

**Authors:** Yuki Numata, Mitsuhito Koizumi, Takao Watanabe, Osamu Yoshida, Yoshio Tokumoto, Kaori Marui, Sho Ishikawa, Masahito Kokubu, Yusuke Okujima, Yoshiki Imamura, Teruki Miyake, Teru Kumagi, Yoichi Hiasa

**Affiliations:** 1 Department of Gastroenterology and Metabology, Ehime University Graduate School of Medicine, Shitsukawa, Toon, Ehime , 791-0295 Japan; 2 Postgraduate Medical Education Center, Ehime University Hospital, Ehime, Japan

**Keywords:** Cell cycle, GADD45A, Pancreatic ductal adenocarcinoma, Protein kinase R, Cancer, Oncogenes

## Abstract

Protein kinase R (PKR) functions both as a promoter and inhibitor in various cancers; however, its role in pancreatic ductal adenocarcinoma (PDAC) remains unclear. We investigated the role of PKR in PDAC. PKR expression in PDAC cell lines was assessed using real-time reverse transcriptase polymerase chain reaction and western blotting. The effect of PKR on cell proliferation was examined using MTS assay. To determine the mechanisms of PKR’s action on PDAC, RNA-sequencing analysis was performed, and the effects of PKR knockdown on cell cycle progression and apoptosis in PDAC cells were examined using flow cytometry. PDAC cell lines transfected with PKR-targeting short interfering RNAs or treated with PKR inhibitors exhibited significantly reduced proliferation. RNA-sequencing analysis revealed substantial upregulation of GADD45A expression upon inhibition of PKR expression. Following PKR silencing, cell cycle analysis showed marked accumulation of cells in G1 phase, consistent with the role of GADD45A as a cell cycle regulator. Proliferation inhibition caused by PKR knockdown was reversed by downregulation of GADD45A, suggesting that PKR and GADD45A interact to regulate PDAC cell growth. PKR promotes PDAC cell proliferation by modulating the cell cycle via regulation of GADD45A expression, demonstrating its potential as a therapeutic target for PDAC.

**Supplementary Information:**

The online version contains supplementary material available at 10.1038/s41598-025-06213-4.

## Introduction

Pancreatic ductal adenocarcinoma (PDAC) has a poor prognosis and is projected to become the second leading cause of cancer-related deaths in the United States by 2040^1^. This bleak outlook is related to the lack of effective drug therapies for PDAC. Therefore, new and effective medications for treating PDAC are urgently needed.

Protein kinase R (PKR), a serine-threonine kinase encoded by *EIF2AK2*, is ubiquitously expressed in human cells^2^. Initially identified to play a key role in the interferon-mediated antiviral response^3^, PKR is activated by various non-viral triggers, such as heat shock proteins, cytokines, mechanical stress, and endoplasmic reticulum stress. It regulates several critical cellular pathways, including mRNA translation, inflammation, stress response, metabolism, cell proliferation, and apoptosis, stimulated by aforesaid factors^4,5^. Recent studies showed that PKR exerts dual roles in cancer, either as a promoter^6–8^ or suppressor^9–11^, depending on the cancer type. This paradoxical effect presumably arises from variations in PKR expression levels and their diverse influence on cellular regulation in different tumor microenvironments^4,12^.

Our studies have revealed that PKR promotes the growth of hepatocellular carcinoma cells infected with the hepatitis C virus^12–14^. However, the role of PKR in PDAC remains unclear. This study was conducted to identify the roles and mechanisms of action of PKR in PDAC through in vitro experiments in PDAC cell lines and analysis of clinical evidence.

## Results

### Expression of PKR is upregulated in PDAC and negatively associated with DFS

**Fig. 1:**
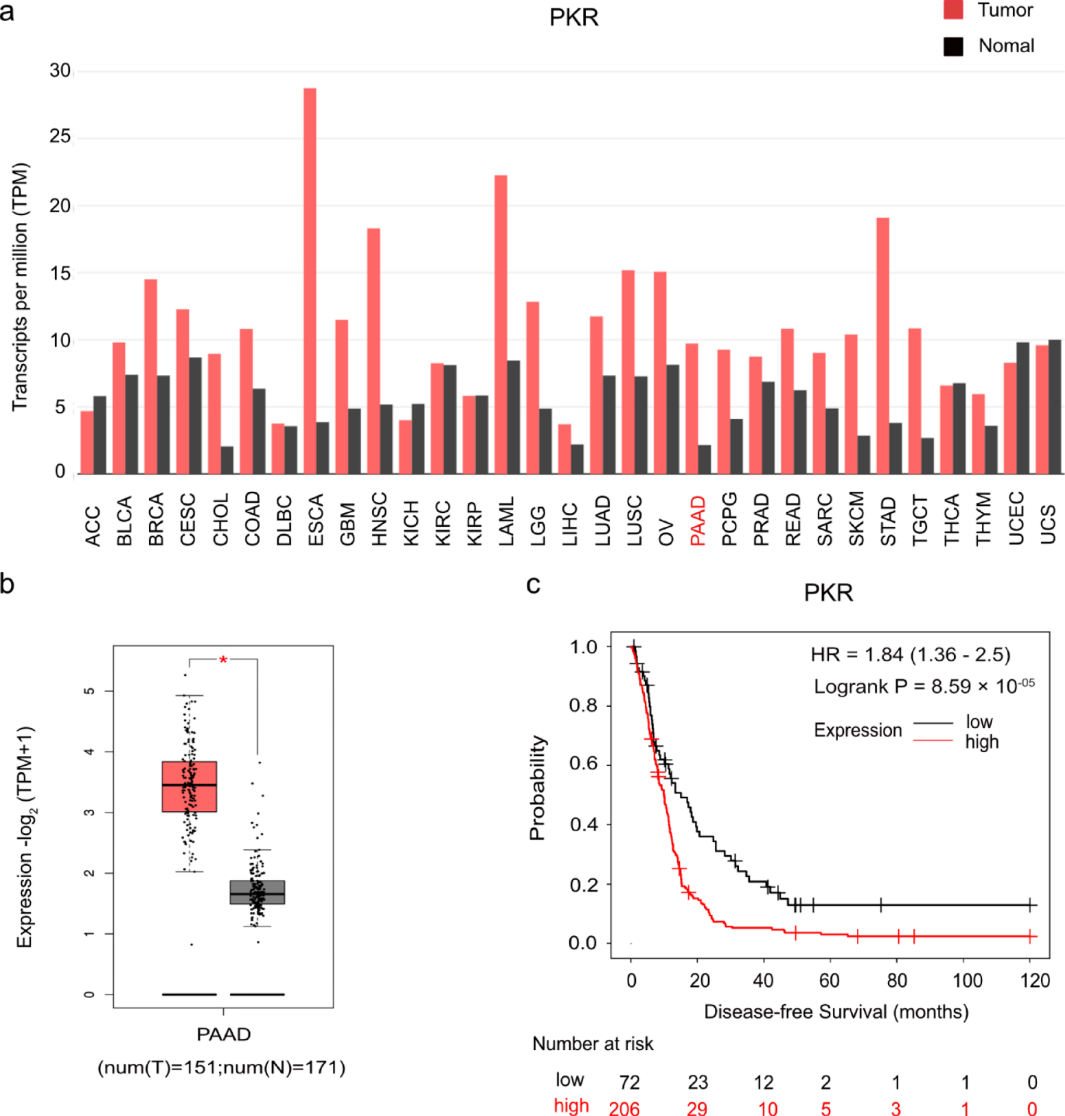
Expression of protein kinase R (PKR) in pancreatic ductal adenocarcinoma (PDAC) tissue and association with disease-free survival (DFS). Expression and clinical significance of PKR in various cancers, particularly PDAC. **(a)** Transcript expression profile of PKR across various tumor types compared to normal tissues. Transcripts per million (TPM) were used as units of expression. The height of the bar represents the median expression of certain tumor type or normal tissue. Red bars represent tumor tissues, whereas gray bars represent normal tissues. **(b)** PKR mRNA level was much higher in patients with pancreatic cancer than in healthy individuals (*P* < 0.01). (c) Kaplan–Meier survival curves showing the clinical significance of PKR expression in patients with PDAC. The panel shows DFS. Patients were divided into high (red curve) and low (gray curve) PKR expression groups.

PKR mRNA expression levels in tumor and normal tissues were compared using Gene Expression Profiling Interactive Analysis (GEPIA; http://gepia.cancer-pku.cn.detail.php) (Fig. 1a and b). Elevated PKR expression in tumor tissues compared to normal tissues was more pronounced in PDAC than in other carcinomas (Fig. 1a). To explore the clinical significance of PKR in PDAC, we performed prognostic analysis using the Kaplan–Meier Plotter database. The results showed that patients with elevated PKR mRNA expression had shorter disease-free survival (DFS) than did those with low PKR expression (*n* = 278; hazard ratio [HR] = 1.84, 95% confidence interval [95% CI] 1.36–2.5, log-rank *p* = 8.59 × 10^−5^) (Fig. 1c).

### Downregulation of PKR expression inhibits the growth of PDAC cells

**Fig. 2:**
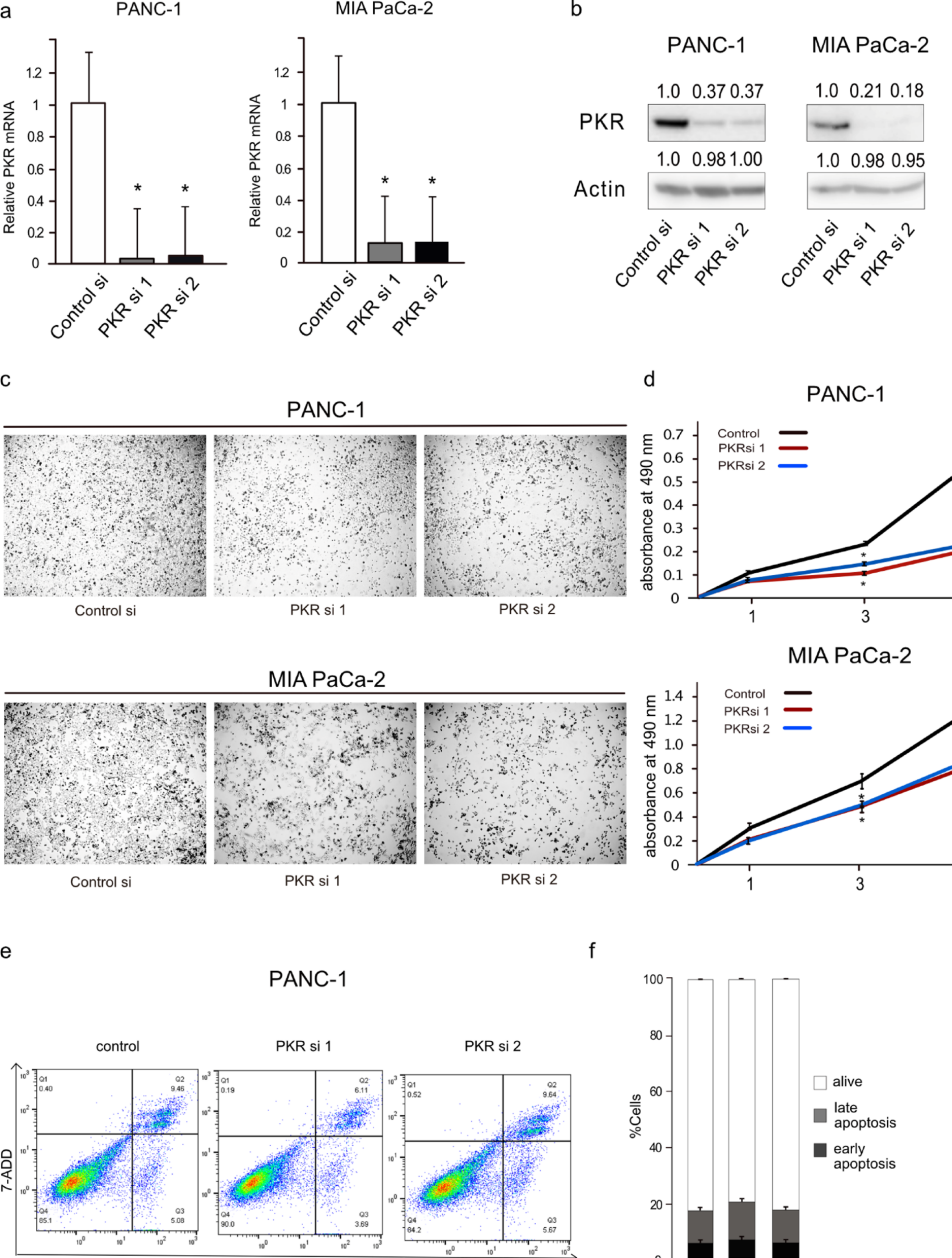
Functional characterization of protein kinase R (PKR) knockdown in pancreatic cancer cell lines PANC-1 and MIA PaCa-2. **(a)** Bar graphs representing relative PKR mRNA expression levels in PANC-1 and MIA PaCa-2 cells post-treatment with control siRNA (Control si) and two different PKR siRNAs (PKR si 1 and PKR si 2). The y-axis displays normalized mRNA expression. Data represent the mean ± standard error of the mean of four replicates. *P < 0.05, vs. control, Student’s *t*-test. **(b)** Western blot analysis showing the protein levels of PKR in PANC-1 and MIA PaCa-2 cells after siRNA treatment. Samples treated with Control si, PKR si 1, and PKR si 2 are depicted. **(c)** Representative images of PANC-1 and MIA PaCa-2 cells on day 3 after control or PKR siRNA transfection. **(d)** Line graphs depicting the proliferation of PANC-1 and MIA PaCa-2 cells, as measured by absorbance at 490 nm, post-siRNA treatment. Data represent the mean ± standard error of the mean of five replicates. *P < 0.05, vs. control, Student’s *t*-test. **(e)** Apoptosis assay of PANC-1 cells treated with *PKR* siRNA, using Annexin V and 7-AAD staining and analyzed using flow cytometry. Dot plots show the proportions of live (Annexin V-negative, 7-AAD-negative), early apoptotic (Annexin V-positive, 7-AAD-negative), and late apoptotic (Annexin V-positive, 7-AAD-positive) cells. **(f)** Quantitative analysis of apoptotic cell distribution from the data in (e). The bar graph shows the percentages of live along with early and late apoptotic cells. Data represent the mean ± standard error of the mean of three replicates. *P < 0.05, vs. control treatment, Student’s *t*-test.

For this study, we selected two pancreatic cancer cell lines with differing levels of PKR expression: PANC-1, which exhibits high PKR expression, and MIA PaCa-2, which exhibits low expression (Supplementary Fig. S1). To investigate the role of PKR in PDAC, both cell lines were transfected with PKR-specific small interfering RNAs (siRNAs). The efficacies of the siRNAs were evaluated using real-time reverse transcription polymerase chain reaction (RT-PCR). The results showed a marked reduction in PKR mRNA expression levels in both cell lines treated with PKR-specific siRNAs compared to that in control cells (Fig. 2a). Western blotting also confirmed knockdown at the protein level, with PKR protein bands less intense in the PKR-specific siRNA-treated groups than in the control group for both cell lines (Fig. 2b). Microscopic observation of the transfected cells revealed differences in cell density and proliferation. PANC-1 and MIA PaCa-2 cells transfected with PKR-specific siRNAs showed reduced density, indicating inhibited growth, with each siRNA treatment, yielding similar suppressive effects (Fig. 2c). Consistently, MTS assays performed at multiple time points showed a marked decrease in the proliferation rates of both cell lines following PKR knockdown (Fig. 2d). In contrast, apoptosis rates were not significantly different between PKR-knockdown and control cells, as indicated by similar percentages of early and late apoptotic cells (Fig. 2e and f). PKR contributes to the proliferative capacity of PDAC cells, with its suppression leading to growth inhibition independent of apoptosis.

### C16 suppressed the proliferation of PDAC cells

**Fig. 3:**
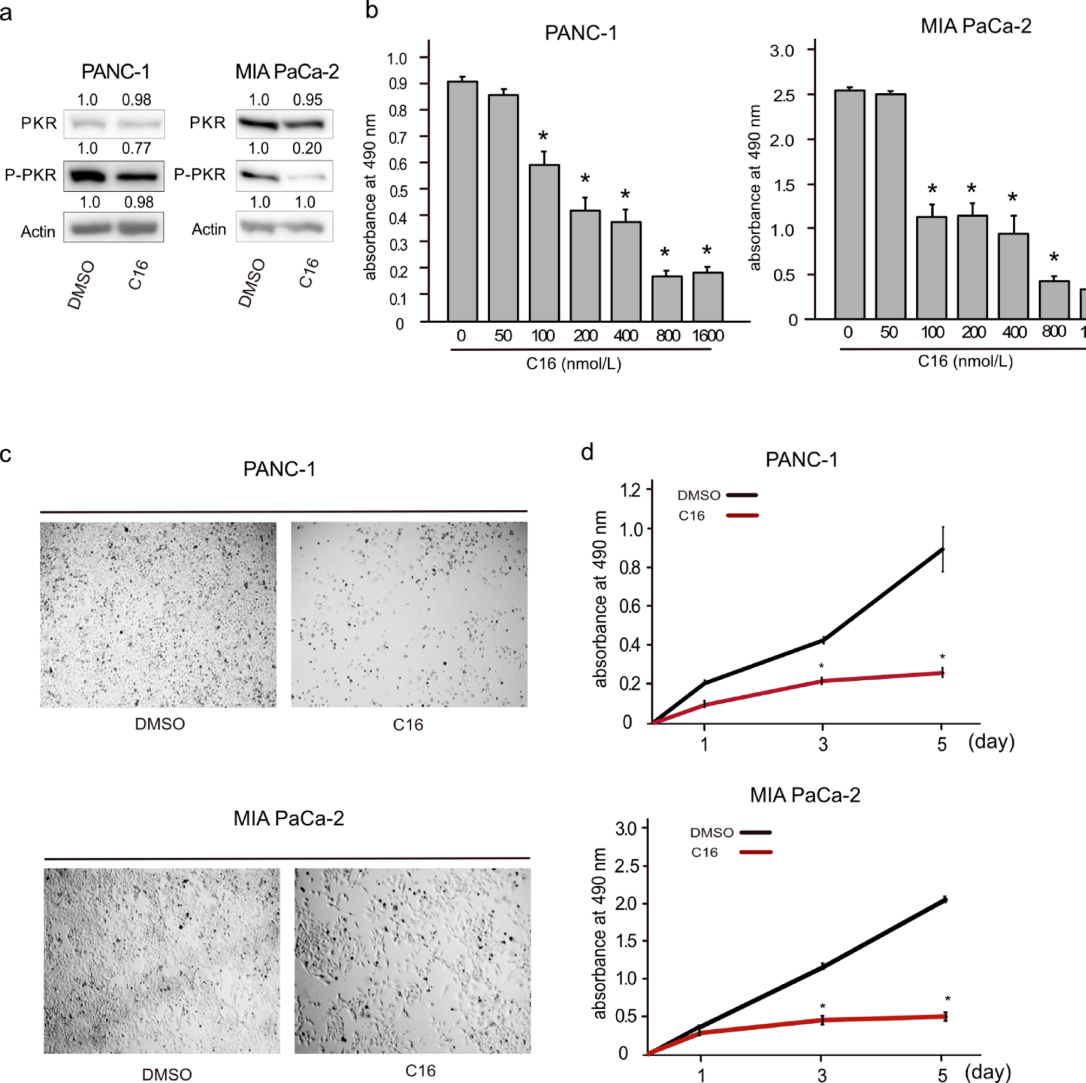
Effects of PKR inhibition in pancreatic ductal adenocarcinoma (PDAC) cells. **(a)** Western blot results displaying the protein levels of PKR and phosphorylated PKR (P-PKR) in PANC-1 and MIA PaCa-2 cells after treatment with dimethyl sulfoxide (DMSO) or a PKR inhibitor (C16) for 72 h. Actin is shown as a loading control. **(b)** Cells were exposed to increasing concentrations of C16, and cell viability was assessed by MTS assay. A concentration-dependent decrease in viability was observed in both cell lines. **(c)** Representative images illustrating the morphology of PANC-1 and MIA PaCa-2 cells after treatment with DMSO or C16 for 3 days. **(d)** Line graphs showing the proliferation of PANC-1 and MIA PaCa-2 cells following treatment with DMSO or 800 nM C16, as measured by absorbance at 490 nm at multiple time points. The y-axis denotes the absorbance values correlating to cell proliferation. Data represent the mean ± standard error of the mean of five replicates. *P < 0.05, vs. DMSO treatment, Student’s *t*-test.

Considering the evident effects of PKR knockdown, further experiments were designed to study the influence of PKR inhibition using the specific inhibitor C16. PANC-1 and MIA PaCa-2 cells were treated with dimethyl sulfoxide (DMSO; control) or C16. Western blotting results showed decreased protein levels of phosphorylated PKR in C16-treated cells (Fig. 3a). Dose-dependent effect of C16 on cell viability in PANC-1 and MIA PaCa-2 cells. Cells were treated with increasing concentrations of C16 for 72 h, and cell viability was evaluated by MTS assay (Fig. 3b). Cells were exposed to increasing concentrations of C16, and cell viability was assessed using the MTS assay. A concentration-dependent decrease in viability was observed in both cell lines. The reduction in cell viability reached a plateau at 800 nM C16. Moreover, microscopic analysis of transfected cells revealed differences in cell density and proliferation, similar to the effects seen following siRNA treatment (Fig. 3c). Substantiating these observations, MTS assays performed at multiple time points derived from absorbance values showed altered proliferation rates in C16-treated cells compared to those of control cells (Fig. 3d). These findings support the pivotal role of PKR in PDAC cell growth. Modulation of PKR activity, either through siRNA-mediated knockdown or pharmacological inhibition, may affect PDAC cell proliferation. Notably, the phosphorylation of eIF2α, a well-known downstream effector of PKR, was not altered by *PKR* knockdown in PANC-1 cells (Supplementary Fig. S2).

### PKR regulates cell cycle in PDAC cells

**Fig. 4:**
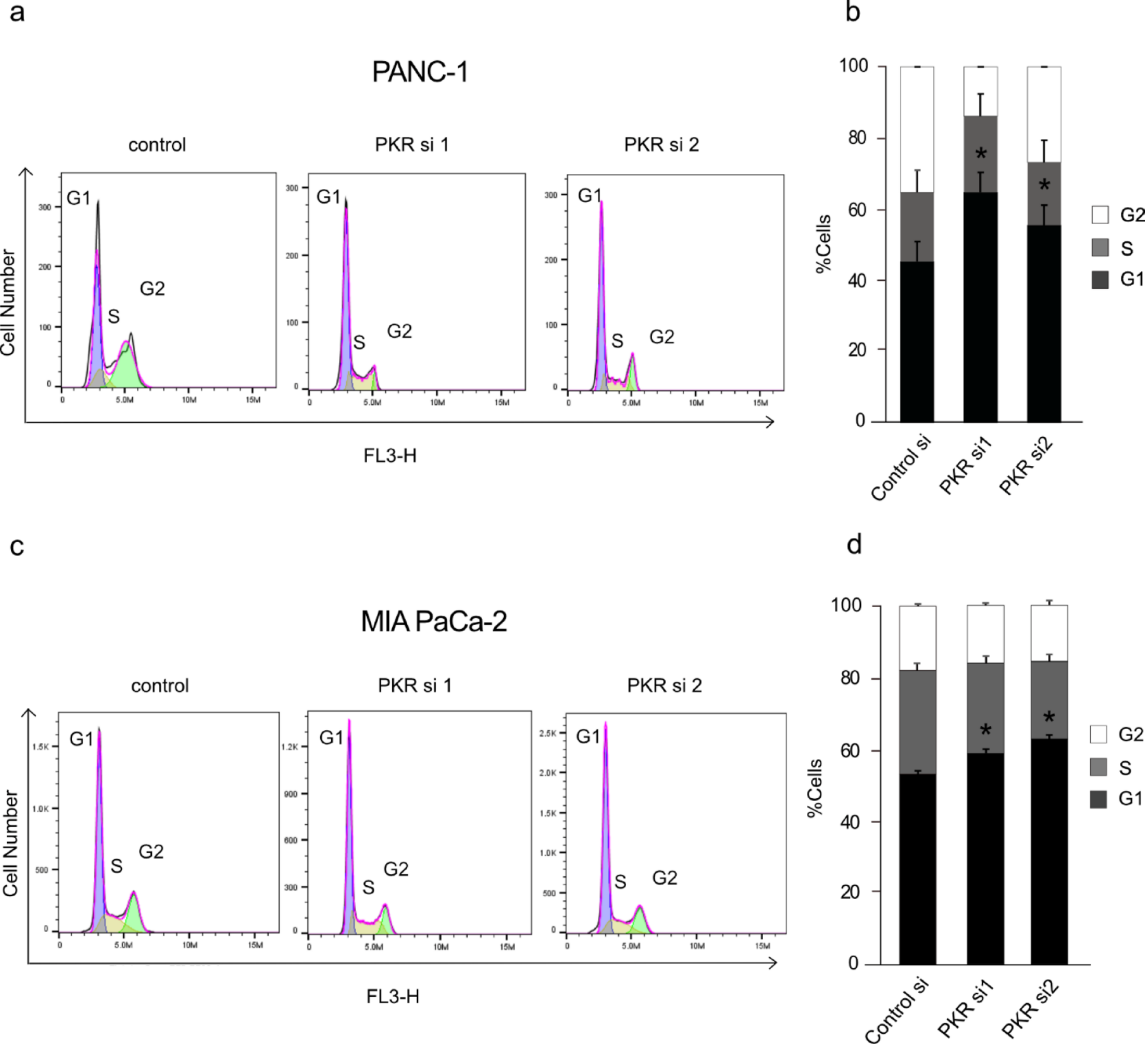
Effect of *PKR* knockdown on cell cycle progression in pancreatic ductal adenocarcinoma (PDAC) cells. **(a**,** b)** Cell cycle analysis of PANC-1 cells transfected with control siRNA or *PKR* siRNA. **(a)** Representative histograms obtained by flow cytometry. G1 phase is shown in purple, S phase in yellow, and G2 phase in green. **(b)** Quantification of cell cycle phase distribution from panel (a). **(c**,** d)** Cell cycle analysis performed in MIA PaCa-2 cells under the same conditions as in (a, b). **(c)** Representative flow cytometry histograms. **(d)** Quantitative analysis of the percentage of cells in each cell cycle phase. Data are presented as mean ± SEM (*n* = 3). Statistical significance was determined using Student’s *t-test* (*p < 0.05, **p < 0.01).

Further, we performed flow cytometry to assess whether *PKR* knockdown affected the cell cycle of PDAC cells. Cell cycle analysis post-*PKR* silencing revealed notable accumulation of cells in the G1 phase (Fig. 4a and b), indicating disrupted cell cycle progression. Similarly, *PKR* knockdown in MIA PaCa-2 cells also led to an increased proportion of cells in the G1 phase (Fig. 4c and d). The observed growth inhibition upon *PKR* knockdown appears to be associated with alterations in cell cycle progression in PDAC cells. In summary, inhibition of PKR activity in PDAC cells predominantly leads to cell cycle arrest in the G1 phase without significantly affecting apoptotic cell death.

### Transcriptional alterations induced by PKR inhibition in PDAC cells

RNA-sequencing (RNA-seq) analysis was performed to determine the detailed molecular mechanisms underlying PKR-mediated PDAC cell proliferation. The Venn diagram was drawn to show the extent of upregulated gene expression following transfection of PANC-1 cells with two different PKR-targeted siRNAs, *PKR*si 1 and *PKR*si 2, as well as after C16 treatment (800 nM) (Fig. S3a). The analysis conducted relative to PANC-1 cells transfected with a control siRNA or treated with DMSO adhered to the selection criteria of a false discovery rate of < 0.05 and fold-change > 1.5. Fourteen genes were upregulated across all treatment groups, suggesting the existence of a core set of PKR-regulated genes (Fig. S3a and Table S1). The counterpart diagram showed that some genes were downregulated under the same experimental conditions (Fig. S3b). Thirty genes were consistently downregulated under all treatment conditions (Fig. S3b and Table S1). Gene set enrichment analysis (GSEA) identified the BIOCARTA_CELLCYCLE_PATHWAY as commonly enriched in both *PKR* knockdown and inhibitor-treated PANC-1 cells, suggesting the consistent involvement of the cell cycle in response to PKR suppression (Fig. S4), although the FDR q-values did not reach statistical significance. Among the upregulated genes was GADD45A, which is implicated in PDAC cell growth and cell cycle^15–17^. Subsequently investigations were conducted to evaluate the relationship between GADD45A and PKR.

### Downregulation of GADD45A reverses the proliferative inhibition induced by PKR knockdown

**Fig. 5:**
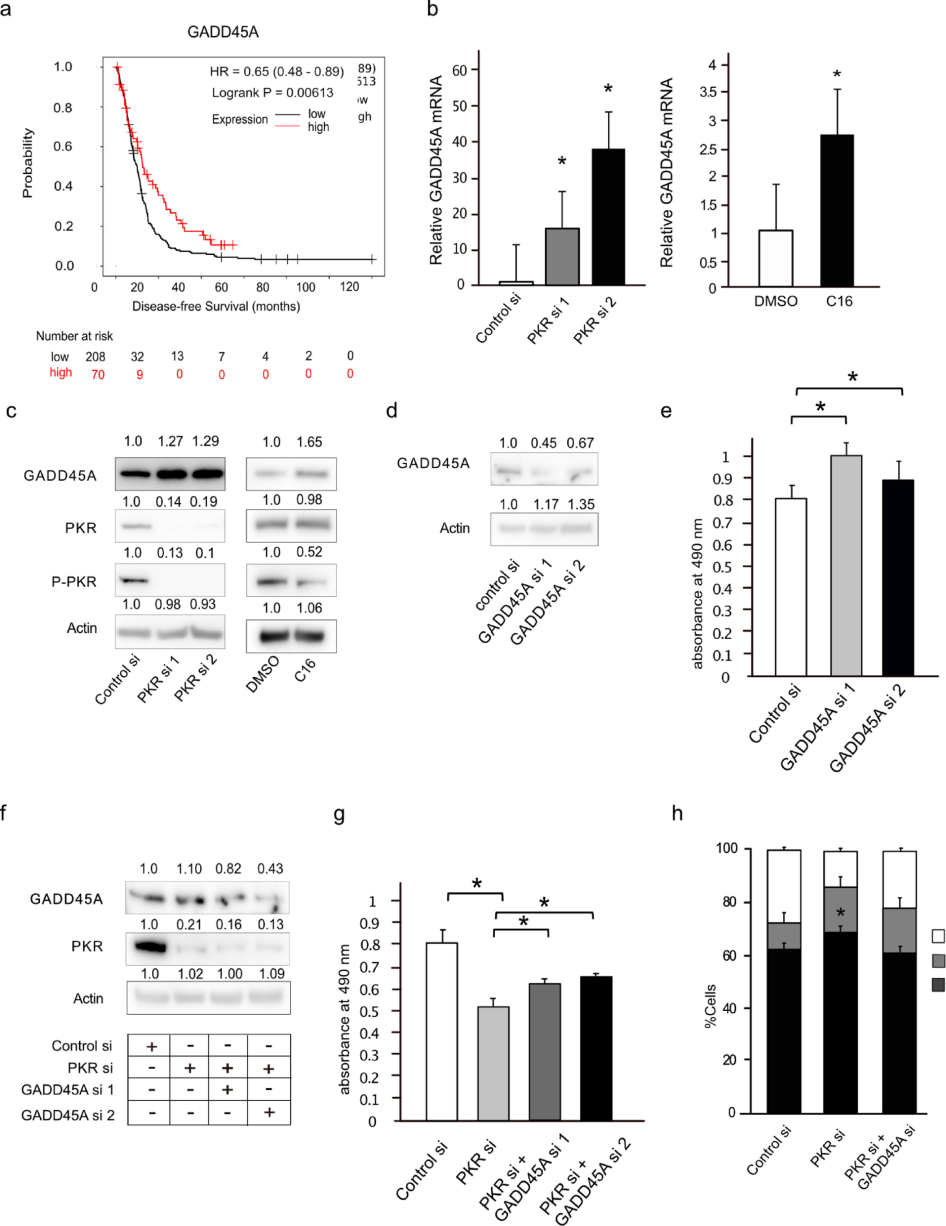
Downregulation of *GADD45A* reverses the proliferative inhibition induced by protein kinase R (*PKR*) knockdown. **(a)** Kaplan–Meier survival curves showing the clinical significance of GADD45A expression in patients with pancreatic ductal adenocarcinoma (PDAC). The panel shows disease-free survival (DFS). Patients were divided into high (red curve) and low (gray curve) groups based on GADD45A expression. **(b)** Quantification of *GADD45A* mRNA levels following transfection with control siRNA, PKR siRNA 1, or PKR siRNA 2 in PANC-1 cells and treated with DMSO or a PKR inhibitor (C16) for 72 h. **(c)** Western blot analysis showing the protein levels of GADD45A, total PKR, and phosphorylated PKR (P-PKR) following transfection with control siRNA, PKR siRNA 1, and PKR siRNA 2 in PANC-1 cells and treatment with DMSO or a PKR inhibitor (C16) for 72 h. Actin was used as a loading control. **(d)** Western blot analysis of GADD45A and PKR protein levels following *GADD45A* siRNA 1 and siRNA2 transfection in PANC-1 cells. Actin was used as a loading control. **(e)** Proliferation assay results (absorbance at 490 nm) of PANC-1 cells transfected with control siRNA, *GADD45A* siRNA1 and siRNA2 on day3 post-transfection. **(f)** Western blot analysis of protein expression following simultaneous knockdown of *PKR* and *GADD45A* in PANC-1 cells. Cells were transfected with control siRNA, *PKR* siRNA, *GADD45A* siRNA, or a combination of siRNAs targeting both *PKR* and *GADD45A*. **(g)** Proliferation assay results (absorbance at 490 nm) of PANC-1 cells co-transfected with control siRNA, PKR siRNAs, and/or GADD45A siRNA on day 3 post-transfection. **(h)** Cell cycle analysis of PANC-1 cells using flow cytometry following transfection with control, PKR, or *GADD45A* siRNAs. Data represent the mean ± standard error of the mean of five replicates. *P < 0.05, Student’s *t*-test.

To explore the clinical significance of GADD45A in PDAC, we performed prognostic analysis using the Kaplan–Meier Plotter database. In contrast to the high PKR expression group (Fig. 1c), the high GADD45A expression group showed prolonged DFS (*n* = 278; HR = 0.65, 95% CI 0.48–0.89) (Fig. 5a). To investigate the interaction between PKR and GADD45A, we analyzed the expression levels of GADD45A in response to PKR knockdown or inhibition. The elevated mRNA and protein levels of GADD45A observed following PKR silencing or inhibition suggested a relationship between the two molecules (Fig. 5b and c). To determine the specific role of GADD45A, we employed siRNA-mediated silencing of GADD45A in PDAC cells. The results revealed a reduction in protein levels, confirming the efficiency of siRNA knockdown (Fig. 5d). The proliferation rate increased post-GADD45A knockdown (Fig. 5e). Following simultaneous knockdown of PKR and GADD45A, western blot analysis was conducted to evaluate the protein expression levels (Fig. 5f). Functionally, MTS proliferation assays revealed that GADD45A downregulation mitigated the inhibition of proliferation caused by PKR knockdown (Fig. 5g). As mentioned above, PKR knockdown increased the percentage of cells in the G1 phase (Fig. 4a and b), and knockdown of both GADD45A and PKR restored the percentage of cells in the G1 phase to those of control cells (Fig. 5h). Consistent with the findings of PKR knockdown, silencing of GADD45A had no apparent effect on apoptosis, as the percentage of apoptotic cells remained unchanged (Supplementary Fig. S5). Thus, the interaction between PKR and GADD45A plays a critical role in the proliferation of PDAC cells by regulating the cell cycle. Moreover, the expression of p21 (encoded by *CDKN1A*), which is known to cooperate with GADD45A in regulating the cell cycle, was altered following *PKR* knockdown (Supplementary Fig. S6).

## Discussion

PKR has an inhibitory effect on tumors^18,19^. However, it was also recently reported to promote tumor growth in breast cancer^6^, melanoma^7^, and lung adenocarcinoma^8^. This dual effect of PKR in different tumors may be related to the varying degrees of PKR expression in different cancer types according to GEPIA data. Particularly, in PDAC, PKR expression is markedly elevated in tumor tissues compared to in non-tumor tissues. Although elevated PKR expression is correlated with significantly poor DFS, indicating its critical role in PDAC progression, its specific role remains unclear.

**Fig. 6:**
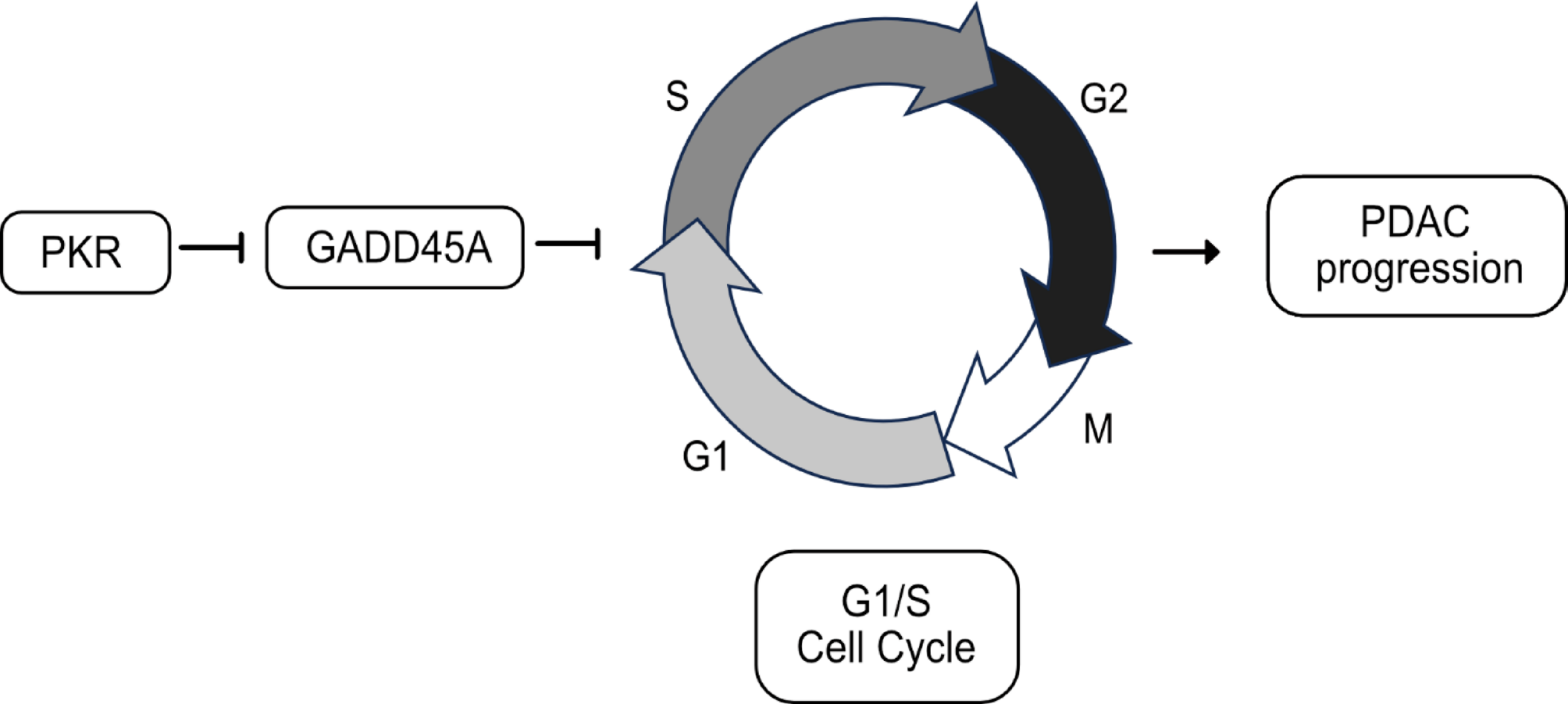
Model of the role of protein kinase R (PKR) in pancreatic ductal adenocarcinoma (PDAC) The PKR and GADD45A pathways regulate the cell cycle in G1/M phase and cell proliferation in PDAC.

We initially demonstrated that PKR downregulation decreases the proliferative potential of PDAC cells, suggesting its involvement in cell proliferation, potentially through cell cycle regulation or apoptosis enhancement. Cell cycle analysis following PKR downregulation showed an increased number of cells in the G1 phase, whereas the number of apoptotic cells was independent of PKR expression. Thus, PKR may be involved in regulating the cell cycle in G1/S phase. Based on this prediction, GADD45A expression was evaluated and found to be predominantly upregulated following both suppression and inhibition of PKR expression. GADD45A is ubiquitously expressed and exerts various functions, such as maintenance of genomic stability, DNA repair, cell cycle control, and apoptosis under diverse cellular stress conditions in multiple cancer types^20^. In PDAC, GADD45A inhibits cell proliferation via apoptosis^15,16^ or cell cycle arrest^17^. GADD45A also interacts with the CDC2/cyclin B1 complex to arrest cells in G2/M phase^17,18,20^ and with p21 to enhance CDKI activity to arrest cells in the G1 phase^21– 22^. In this study, the simultaneous knockdown of PKR and GADD45A reversed the decreased growth of PDAC cells caused by PKR knockdown. Furthermore, PKR inhibition increased the number of cells in the G1 phase. Thus, PKR regulates PDAC cell growth by modulating the cell cycle through GADD45A (Fig. 6). The association between PKR and GADD45A has not been reported previously. A recent study showed that PERK (PKR-like endoplasmic reticulum kinase), an eIF2 phosphorylated kinase similar to PKR, enhances GADD45A expression, causing apoptotic cell death and contributing to the suppression of PDAC^16^. In our study, the phosphorylation level of eIF2α remained unchanged after *PKR* knockdown or treatment with the *PKR* inhibitor (Fig. S2), suggesting that changes in the proliferative potential of PDAC cells through cell cycle regulation are associated with mechanisms independent of the eIF2α pathway. We hypothesized that the cell cycle regulatory mechanism of GADD45A activates a pathway other than apoptosis regulation. The observed upregulation of GADD45A expression following *PKR* knockdown or inhibition suggests PKR may play a role as a transcriptional regulator of GADD45A. While PKR is primarily known for its role in translational inhibition through phosphorylation of eIF2α, it also reportedly participates in transcriptional regulation^4^. In particular, NF-κB, whose activation is promoted by PKR, suppresses the expression of GADD45A. Further investigation is warranted to elucidate these regulatory mechanisms^23^. One potential downstream effector is p21. Our data demonstrate that inhibition of PKR significantly upregulates the expression of *CDKN1A*, the gene encoding p21 (Fig. S6 and Table S1). p21 is a key mediator of cell cycle arrest and functions downstream of p53, acting in concert with GADD45A. p21 is a representative factor for cell cycle arrest and acts downstream of p53 in concert with GADD45A^24^. Thus, PKR, GADD45A, and p21 are interrelated and regulate the cell cycle. The proliferation of PDAC cells upon simultaneous knockdown of *PKR* and *GADD45A* did not fully return to the level observed with *GADD45A* knockdown alone, despite GADD45A expression being suppressed below the baseline. This suggests that PKR may also regulate cell proliferation through mechanisms independent of GADD45A, possibly involving alternative pathways or factors yet to be identified. The PKR inhibitor C16, used in this study, is an ATP-binding site-directed small molecule that inhibits PKR autophosphorylation^25^. C16 suppresses tumor proliferation in hepatocellular carcinoma^26^. PKR inhibitors also inhibit the proliferation of pancreatic cancer cell lines; they have been safely administered in murine models^26,27^ and are expected to be used in humans. However, in vivo studies using PKR inhibitors in humans have not been conducted.

In addition to its well-established roles in translational regulation and cell cycle control, PKR is also a key modulator of innate immune responses. It is activated by various stressors, including viral double-stranded RNA, leading to downstream signaling through pathways such as NF-κB and interferon regulatory factors (IRFs), which play crucial roles in inflammation and antiviral defense^3–5^. This immune-regulatory function of PKR has also been implicated in tumor immunity. PKR activation can lead to the production of pro-inflammatory cytokines and type I interferons, shaping the tumor immune microenvironment^4^. Conversely, aberrant activation of PKR may promote immune evasion by regulating immunosuppressive cytokine production or modulating antigen presentation^5^.

Although our study focused primarily on the role of PKR in PDAC cell cycle regulation via GADD45A, it is plausible that PKR may also influence PDAC progression by modulating antitumor immune responses. Given that PDAC is characterized by an immunosuppressive microenvironment, future investigations are warranted to explore whether PKR contributes to immune evasion mechanisms in PDAC, potentially through NF-κB activation or modulation of interferon signaling. Such insights could uncover the dual role of PKR in PDAC, both as a regulator of cell proliferation and a modulator of the tumor immune landscape, further supporting its potential as a therapeutic target.

In conclusion, our results suggest that suppression of PKR inhibits the proliferation of PDAC cells by arresting the cell cycle via GADD45A. Thus, PKR shows potential as a therapeutic target for the treatment of PDAC. Moreover, the PKR inhibitor should be evaluated in humans to establish its clinical applicability in PDAC.

## Methods

### Cell culture

The pancreatic cancer cell lines PANC-1 and MIA PaCa-2 were purchased from American Type Culture Collection (Manassas, VA, USA) and cultured in Dulbecco’s modified Eagle’s medium (Thermo Fisher Scientific, Waltham, MA, USA) supplemented with 10% fetal bovine serum (Thermo Fisher Scientific) and 1% penicillin. Cells were incubated in a 37 ℃ humidified incubator with 5% CO_2_.

### Gene expression analysis in cancer cell lines

Gene expression data for *EIF2AK2* (PKR) was retrieved from the DepMap Portal database (Expression Public 24Q4 release). The analysis included 64 pancreatic adenocarcinoma cell lines selected from a total of 2,015 cancer cell lines available in the database, allowing for focused evaluation of EIF2AK2 expression patterns in this specific cancer type. Log_2_-transformed TPM + 1 (transcripts per million) values were used to measure gene expression levels. The data was ranked based on expression levels, with the x-axis representing the rank of each cell line, going from lowest to highest expression.

### Kaplan–Meier plotter database

Kaplan**–**Meier Plotter (http://kmplot.com/analysis), an online database, was used to assess the correlation between gene expression and survival^28^. We used the Kaplan–Meier Plotter tool to analyze the relationship between PKR mRNA expression and DFS in PDAC. Similarly, the correlation between growth arrest and DNA damage-inducible 45 alpha (GADD45A) mRNA levels and DFS was analyzed. The results are presented as HRs, 95% CIs, and computed log-rank p-values.

### Gene expression profiling interactive analysis dataset

GEPIA (http://gepia.cancer-pku.cn/) is an interactive online analysis tool that includes mRNA expression data for 9,736 tumor tissues from The Cancer Genome Atlas and 8,587 normal tissues from the Genotype-Tissue Expression database^29^. GEPIA was used to compare the expression levels of PKR between pancreatic cancer and normal pancreatic tissues.

### Western blotting

Proteins were extracted with RIPA buffer comprising 10 mmol/L Tris, PH 7.4, 150 mmol/L NaCl, 0.5% v/v NP-40, and 1% w/v sodium dodecyl sulfate. Protein (20 µg) was loaded onto 4–12% Bis-Tris gels (Thermo Fisher Scientific) and then transferred to Immobilon-P membranes (Millipore, Billerica, MA, USA). The membranes were incubated with the relevant antibodies (1:1000 dilutions) (Table S2) for 10 h at 4 ℃ and then incubated with horseradish peroxidase-conjugated secondary antibodies (GENA934-1ML; Cytiva, Marlborough, MA, USA, 1:10000) and anti-mouse immunoglobulin G (NA934-1ML; Cytiva). Signals were detected using an ECL Prime Kit (Cytiva) and ImageQuant LAS 4000 system (GE Healthcare, Chicago, IL, USA). The density of the bands was quantified using ImageJ software (National Institutes of Health, Bethesda, MD, USA).

### Transfection of SiRNA

We used PKR-specific siRNA1 (5′-GCA GAU ACA UCA GAG AUA A-3′) and siRNA2 (5′-GAU CUU AAG CCA AGU AAU A-3′), GADD45A-specific siRNA (5′-UAA UCU CCC UGA ACG GUG A-3′), and control siRNA (Horizon Discovery, Cambridge, UK). PANC-1 and MIA PaCa-2 cells were transfected with 50 pM siRNA using RNAiMAX (Thermo Fisher Scientific). After 72 h, RNA and proteins were extracted from the cells.

### RNA extraction, cDNA synthesis, and real-time reverse transcription polymerase chain reaction

Total RNA was extracted from PANC-1 and MIA PaCa-2 cells using TRIzol reagent (Thermo Fisher Scientific). RNA was reverse-transcribed using a High-Capacity cDNA Reverse Transcription Kit with an RNase inhibitor (Applied Biosystems, Foster City, CA, USA). Real-time PCR was performed on a LightCycler 480 (Roche, Basel, Switzerland) with SYBR Green I (Roche, BSL, Switzerland). The primer sequences used to amplify the human genes are listed in Table S3. Glyceraldehyde 3-phosphate dehydrogenase (*GAPDH*) was used as an internal reference gene. Relative changes in mRNA levels were determined by relative quantification using the 2^−ΔΔCt^ method.

### Chemicals

The PKR inhibitor C16 was purchased from Merck (Kenilworth, NJ, USA) and solubilized in DMSO. The final concentration of C16 was adjusted to 800 nM. DMSO was used as a control.

### Cell proliferation assay

Cell viability was quantified using the MTS assay (Promega, Madison, WI, USA). Cells were seeded at 2,000 per well in 96-well plates and transfected with siRNA the following day. At 72 h post-transfection, the cells were treated with MTS reagent and incubated for 2 h. The absorbance at 490 nm was recorded using a plate reader (Asys Expert 96; Biochrom, Cambridge, UK).

### Cell cycle analysis

Cell Cycle Assay Solution Deep Red (Deep Red; Dojindo Molecular Technologies, Kumamoto, Japan) was used to detect the distribution of cells at different phases of the cell cycle. Cells were seeded at a density of 1 × 10^6^ cells/dish in 10-cm dishes. At 72 h after siRNA transfection, the samples were washed with PBS, resuspended in PBS, and stained with Cell Cycle Assay Solution Deep Red followed by incubation at 37 ℃ for 15 min according to the manufacturer’s protocol. The samples were analyzed using flow cytometry (Gallios flow cytometer; Beckman Coulter, Brea, CA, USA).

### Apoptosis assay

The apoptosis assay was performed using Annexin V (BD Biosciences) and 7-amino-actinomycin D (7-AAD) (Immunostep, Salamanca, Spain). Cells were seeded at a density of 1 × 10^6^ cells/dish in 10-cm dishes. At 72 h after siRNA transfection, the cells were detached using 0.05% trypsin and washed twice with FCM buffer [0.5% bovine serum albumin, 0.01% NH_2_ (pH 7.3)]. The samples were resuspended in 1× annexin-binding buffer and incubated with 5 µL Annexin V-FITC and 5 µL 7-AAD for 15 min at room temperature while avoiding exposure to light. Finally, the stained samples were analyzed using a Guava EasyCyte Flow Cytometer (Gallios flow cytometer; Beckman Coulter).

### RNA-seq and data analyses

Total RNA was collected from PANC-1 cells 72 h after transfection with control, PKR siRNA 1, or PKR siRNA 2. The integrity of the isolated RNA was verified using an Agilent 2100 Bioanalyzer (Agilent Technologies, Santa Clara, CA, USA) and an RNA6000 Nano Kit (Agilent Technologies). RNA-seq libraries were prepared using the NEBNext Ultra II Directional RNA Library Prep Kit from Illumina (San Diego, CA, USA), NEBNext Poly(A) mRNA Magnetic Isolation Module, and NEBNext Multiplex Oligos (New England Biolabs, Ipswich, MA, USA). The quality of the RNA-seq libraries was verified, and the average size of the libraries was calculated using a 2100 Bioanalyzer and Agilent DNA1000 kit (Agilent Technologies). The qPCR-based libraries were quantified using a Kapa Library Quantification Kit (Illumina). Paired end reads (75 bp) were sequenced using the MiSeq Reagent Kit V3 for 150 cycles on a MiSeq system (Illumina). Mapping to the human genome data hg38 was performed using TopHat (https://ccb.jhu.edu/software/tophat/index.shtml), and sequence reads were assigned to genomic features using feature counts^30^. The differential expression analysis tool TCC (Bioconductor.org) was used to normalize each sample before determining the differences in expression between control siRNA (*n* = 3), PKR siRNA 1 (*n* = 3), and PKR siRNA 2 (*n* = 3) using the tmm package in R (https://www.r-project.org/). The same method was used for RNA-seq analysis of RNA extracted 72 h after treatment of PANC-1 cells with DMSO or C16 (800 nM). Raw and processed data were submitted to the Gene Expression Omnibus (GSE285947) database. GSEA was conducted using GSEA software^31^.

### Statistical analyse*s*

All statistical analyses were performed using JMP version 13.2 (SAS Institute, Cary, NC, USA). Data are expressed as the mean and standard deviation. Statistical differences were analyzed using Student’s *t*-test or Wilcoxon test. Correlations between two variables were evaluated using Pearson’s correlation coefficients. Statistical significance was set at *P* < 0.05, based on two-tailed tests.

## Electronic supplementary material

Below is the link to the electronic supplementary material.

Supplementary Material 1

## Data Availability

The datasets generated and/or analysed during the current study have been deposited in the Gene Expression Omnibus (GEO) repository under the accession number GSE285947.
