# Supplementary material for: Protein kinase R regulates pancreatic ductal adenocarcinoma progression by modulating the cell cycle via GADD45A
Source: Sci Rep. 2025 Jul 10;15:24966. doi: 10.1038/s41598-025-06213-4 (PMC12246039; doi:10.1038/s41598-025-06213-4)
Supplement: Supplementary file 1 — Supplementary Material 1 [file 41598_2025_6213_MOESM1_ESM.docx]

**Supplementary Information**

**Protein kinase R regulates pancreatic ductal adenocarcinoma progression by modulating the cell cycle via *GADD45A***

Yuki Numata, Mitsuhito Koizumi, Takao Watanabe, Osamu Yoshida, Yoshio Tokumoto, Kaori Marui, Sho Ishikawa, Masahito Kokubu, Yusuke Okujima, Yoshiki Imamura, Miyake Teruki, Teru Kumagi, Yoichi Hiasa

S**upplementary Table S1. Genes commonly upregulated under three conditions**

| Ensembl Gene ID | Gene Symbol |
| --- | --- |
| ENSG00000154380 | *ENAH* |
| ENSG00000123505 | *AMD1* |
| ENSG00000011052 | *NME1-NME2* |
| ENSG00000100292 | *HMOX1* |
| ENSG00000155380 | *SLC16A1* |
| ENSG00000075426 | *FOSL2* |
| ENSG00000162772 | *ATF3* |
| ENSG00000116717 | *GADD45A* |
| ENSG00000144597 | *EAF1* |
| ENSG00000175197 | *DDIT3* |
| ENSG00000084070 | *SMAP2* |
| ENSG00000124762 | *CDKN1A* |
| ENSG00000172071 | *EIF2AK3* |
| ENSG00000090674 | *MCOLN1* |

***Three conditions:**

PANC-1 cells were either transfected with two distinct PKR-targeting siRNAs (PKRsi 1, PKRsi 2) or treated with varying concentrations of the PKR inhibitor (800 nM) for 72 h.

**Genes commonly downregulated under three conditions**

| Ensembl Gene ID | Gene Symbol |
| --- | --- |
| ENSG00000185033 | *SEMA4B* |
| ENSG00000142798 | *HSPG2* |
| ENSG00000162337 | *LRP5* |
| ENSG00000185215 | *TNFAIP2* |
| ENSG00000099282 | *TSPAN15* |
| ENSG00000103222 | *ABCC1* |
| ENSG00000108846 | *ABCC3* |
| ENSG00000109790 | *KLHL5* |
| ENSG00000149527 | *PLCH2* |
| ENSG00000100403 | *ZC3H7B* |
| ENSG00000006062 | *MAP3K14* |
| ENSG00000186260 | *MRTFB* |
| ENSG00000100311 | *PDGFB* |
| ENSG00000174705 | *SH3PXD2B* |
| ENSG00000186815 | *TPCN1* |
| ENSG00000166920 | *C15orf48* |
| ENSG00000103490 | *PYCARD* |
| ENSG00000170412 | *GPRC5C* |
| ENSG00000126464 | *PRR12* |
| ENSG00000164985 | *PSIP1* |
| ENSG00000187325 | *TAF9B* |
| ENSG00000163898 | *LIPH* |
| ENSG00000039139 | *DNAH5* |
| ENSG00000125746 | *EML2* |
| ENSG00000188636 | *RTL6* |
| ENSG00000006210 | *CX3XL1* |
| ENSG00000142197 | *DOP1B* |
| ENSG00000134575 | *ACP2* |
| ENSG00000111679 | *PTPN6* |
| ENSG00000162804 | *SNED1* |

***Three conditions:**

PANC-1 cells were either transfected with two distinct PKR-targeting siRNAs (PKRsi 1, PKRsi 2) or treated with varying concentrations of the PKR inhibitor (800 nM) for 72 h.

S**upplementary Table S2. Antibodies used in this study**

| Antibody | Company | Protocol |
| --- | --- | --- |
| PKR | Cell Signaling Technology, #3072 | WB, IP |
| PKR (phosphor T446) | Abcam, ab32036 | IHC, WB, IP |
| GADD45A | Abcam, ab180768 | WB |
| β-actin | Proteintech, 60008-1-1g | IHC, WB, IF, FC |

IHC, immunohistochemistry. WB, western blotting. IP, immunoprecipitation

IF, immunofluorescence. FC, flow cytometry

S**upplementary Table S3. Primer sequences used for real-time PCR**

| *EIF2AK2* | Forward primer | TGTTGGGATGGATTTGATTAATG |
| --- | --- | --- |
|  | Reverse primer | GAAAAGGCACTTAGTCTTTGACCT |
| *GADD45A* | Forward primer | GACTTTGGAGGAATTCTCGGCTG |
|  | Reverse primer | CTTCGTACACCCCGACAGTGA |
| *GAPDH* | Forward primer | AGCCACATCGCTCAGACAC |
|  | Reverse primer | GCCCAATACGACCAAATCC |

Supplementary Fig. S1 PKR expression in pancreatic cancer cell lines

**(a)** Waterfall plot showing the ranked expression of EIF2AK2 (PKR) across 64 pancreatic adenocarcinoma cell lines from the DepMap Portal database (Expression Public 24Q4). The y-axis displays log_2_(TPM+1) expression values, while the x-axis shows the rank order of cell lines based on expression level. Each circle represents a distinct cancer cell line. Two cell lines of interest, PANC1 and MIAPACA2, are highlighted in darker blue and labeled. **(b)** PKR expression in PANC-1 and MIA PaCa-2 cells was confirmed by western blot analysis.

Supplementary Fig. S2. Phosphorylation of eIF2α after transfection with PKR-specific siRNA or treatment with a protein kinase R (PKR) inhibitor

Western blot analysis showing the protein levels of phosphorylated EIF2α (P-EIF2α) in PANC-1 cells after treatment with the PKR inhibitor (C16) for 72 h.

Supplementary Fig. S3. Transcriptional profiling of pancreatic ductal adenocarcinoma (PDAC) cells with protein kinase R (PKR) knockdown or PKR inhibition

**(a)** Venn diagram showing the overlap of upregulated genes in PANC-1 cells subjected to different treatments. Cells were either transfected with two distinct PKR-targeting siRNAs (PKRsi 1, PKRsi 2) or treated with varying concentrations of the PKR inhibitor (800 nM) for 72 h. Gene upregulation was determined relative to control siRNA-transfected or DMSO-treated cells under the criteria of false discovery rate < 0.05 and fold-change > 1.5. The panel lists the 14 genes upregulated under all treatment conditions. **(b)** Corresponding Venn diagram shows genes that were downregulated under the same treatment conditions described above. This panel revealed specific subsets of genes suppressed by each treatment and highlighted 30 genes that were consistently downregulated.

Supplementary Fig. S4. GSEA enrichment plots of the BIOCARTA_CELLCYCLE_PATHWAY in pancreatic ductal adenocarcinoma (PDAC) cells following protein kinase R (PKR) knockdown or PKR inhibition

**(a)** Gene set expression analysis (GSEA) plot of PANC-1 cells transfected with two different PKR-targeted siRNAs. **(b)** GSEA plot of PANC-1 cells treated with the PKR inhibitor C16 (800 nM). NES normalized enrichment score, FDR false discovery rate.

Supplementary Fig. S5. Analysis of apoptosis in PANC-1 cells following *GADD45A* knockdown.

Apoptosis assay of PANC-1 cells treated as in *GADD45A* siRNA using Annexin V and 7-AAD staining and analyzed using flow cytometry. The bar graph shows the percentages of live along with early and late apoptotic cells. Data represent the mean ± standard error of the mean of three replicates. *P < 0.05, vs. control treatment, Student’s *t*-test.

Supplementary Fig. S6. Silencing of protein kinase R (PKR) leads to CDKN1A upregulation

1. Panc-1 cells were transfected with control, *PKR* siRNA1, or *PKR* siRNA2. *CDKN1A* mRNA

expression was quantified using RT-PCR. B. Panc-1 cells were treated with DMSO or C16 (800 nM) for 72 h, after which the RNAs were analyzed. *CDKN1A* mRNA expression was measured using RT-PCR. Data represent the mean ± standard error of the mean of five replicates. *P < 0.05, vs. DMSO treatment, Student’s *t*-test. **b)** Panc-1 cells were treated with DMSO or C16 (800 nM) for 72 h, after which the RNAs were analyzed. *CDKN1A* mRNA expression was measured using RT-PCR. Data represent the mean ± standard error of the mean of five replicates. *P < 0.05, vs. DMSO treatment, Student’s *t*-test.

Supplementary Fig. S7. Original blots for Fig. 2b.

Supplementary Fig. S8. Original blots for Fig. 3a.

Supplementary Fig. S9. Original blots for Fig. 5c.

Supplementary Fig. S10. Original blots for Fig. 5d and 5e.

Supplementary Fig. S11. Original blots for Supplementary Fig. S2.
